# A Comparative Atlas-Based Recognition of Mild Cognitive Impairment With Voxel-Based Morphometry

**DOI:** 10.3389/fnins.2018.00916

**Published:** 2018-12-06

**Authors:** Zhuqing Long, Jinchang Huang, Bo Li, Zuojia Li, Zihao Li, Hongwen Chen, Bin Jing

**Affiliations:** ^1^Medical Apparatus and Equipment Deployment, Nanfang Hospital, Southern Medical University, Guangzhou, China; ^2^School of Biomedical Engineering, Capital Medical University, Beijing, China; ^3^Department of Acupuncture and Minimally Invasive Oncology, Beijing University of Chinese Medicine Third Affiliated Hospital, Beijing, China; ^4^Department of Traditional Chinese Medicine, Beijing Chest Hospital, Capital Medical University, Beijing, China; ^5^Beijing Tuberculosis and Thoracic Tumor Research Institute, Beijing, China

**Keywords:** mild cognitive impairment, brain parcellation, automated anatomical labeling atlas, brainnetome atlas, voxel-based morphometry

## Abstract

An accurate and reliable brain partition atlas is vital to quantitatively investigate the structural and functional abnormalities in mild cognitive impairment (MCI), generally considered to be a prodromal phase of Alzheimer’s disease. In this paper, we proposed an automated structural classification method to identify MCI from healthy controls (HC) and investigated whether the classification performance was dependent on the brain parcellation schemes, including Automated Anatomical Labeling (AAL-90) atlas, Brainnetome (BN-246) atlas, and AAL-1024 atlas. In detail, structural magnetic resonance imaging (sMRI) data of 69 MCI patients and 63 HC matched well on gender, age, and education level were collected and analyzed with voxel-based morphometry method first, then the volume features of every region of interest (ROI) belonging to the above-mentioned three atlases were calculated and compared between MCI and HC groups, respectively. At last, the abnormal volume features were selected as the classification features for a proposed support vector machine based identification method. After the leave-one-out cross-validation to estimate the classification performance, our results reported accuracies of 83, 92, and 89% with AAL-90, BN-246, and AAL-1024 atlas, respectively, suggesting that future studies should pay more attention to the selection of brain partition schemes in the atlas-based studies. Furthermore, the consistent atrophic brain regions among three atlases were predominately located at bilateral hippocampus, bilateral parahippocampal, bilateral amygdala, bilateral cingulate gyrus, left angular gyrus, right superior frontal gyrus, right middle frontal gyrus, left inferior frontal gyrus, and left precentral gyrus.

## Introduction

Mild cognitive impairment (MCI), which represents the transition state between normal aging and the early changes related to Alzheimer’s disease (AD) (Han et al., 2011; Wang et al., 2015; Khazaee et al., 2016, 2017), is characterized by intellectual and cognitive deficits, memory complaints, and behavioral disturbances (Zhang et al., 2012; Beheshti et al., 2017), and generally regarded as a prodromal phase of AD (Long et al., 2018). Overall, the prevalence of MCI in the elderly is 19%, and nearly half of them will evolve to AD within 3 to 5 years (Long et al., 2016). Increasing attention from neurologists, neuroscientists, and neuroradiologists has been paid to MCI due that early intervention before irreversible brain tissue damage is crucial for efficient AD treatments (Davatzikos et al., 2008a). Therefore, accurate MCI identification methodologies that could serve as non-invasive surrogates for these pathologic examinations are desperately needed, which may provide additional insights into the clinical diagnosis of MCI.

Structural magnetic resonance imaging (sMRI) has been prevalently utilized to characterize differences in shape and neuroanatomical configuration in MCI and AD because it could provide visualization of the macroscopic tissue atrophy caused by the cellular changes underlying MCI and AD (Desikan et al., 2009). By analyzing the sMRI data with voxel-based morphometry (VBM) method, which is utilized to assess the structure of the whole brain with voxel-by-voxel comparisons between groups in an anatomically unbiased way (Ashburner and Friston, 2000), many prior studies found that the atrophic brain regions mainly lay in the medial temporal lobe containing hippocampus, parahippocampal, and amygdala in MCI and AD (Baron et al., 2001; Hirata et al., 2005). In addition, some studies employed sMRI data to identify MCI or AD from healthy controls (HC) by extracting structural characteristics such as voxel-wise volume (Fan et al., 2007; Davatzikos et al., 2008a,b; Klöppel et al., 2008; Magnin et al., 2009; Beheshti and Demirel, 2016) and vertex-based cortical thickness (Lerch et al., 2008; Eskildsen et al., 2013; Dimitriadis et al., 2018), and the classifying accuracies varied largely from 58% to 100%, which indicated that the discriminative diagnoses of MCI and AD with sMRI data need to be continued.

From the previous voxel-based MCI or AD discrimination studies, these studies could be roughly classified into two categories, data-driven adaptive characteristic extraction methods (Misra et al., 2009; Davatzikos et al., 2011) and atlas-based partition characteristic extraction methods with a predefined brain atlas (Cuingnet et al., 2011; Cho et al., 2012; Liu et al., 2015). The former method was not easy to interpret anatomically because each region of interest (ROI) obtained from the input data may involve in many anatomical regions simultaneously. In contrast, the latter could better extract the classification features with a good anatomical interpretability. At present, the automated anatomical labeling (AAL-90) atlas is the most popular atlas, which has been widely employed to identify kinds of psychological disorders in recent years (Dai et al., 2012; Wee et al., 2012; Zeng et al., 2012). Besides, some other atlases were also proposed, such as AAL-1024 atlas (Zhang et al., 2011; Wu et al., 2013) and the novel connectional architecture based brainnetome (BN-246) atlas (Fan et al., 2016). Different brain atlases lead to different partitions in terms of the number of regions and the size and location of these regions in the brain (Asim et al., 2018). Till now, few studies compared the classification performance with different atlases (Mesrob et al., 2009; Ota et al., 2014, 2015; Asim et al., 2018), and no study has utilized the BN-246 atlas to identify MCI from HC subjects with structural data. Moreover, it remains unknown whether BN-246 atlas would perform better compared with the two above-mentioned atlases in identifying MCI patients from HC subjects. Also, it is intriguing whether better accuracy could be acquired by using the shared features extracted from three atlases.

In this paper, we proposed an automated classification method to identify MCI from HC and aimed to investigate whether the classification performance was dependent upon the brain parcellation schemes. To accomplish this goal, we first analyzed the sMRI data with VBM analysis, and then the volume features of every ROI in the above-mentioned three atlases were calculated and compared between MCI and HC groups, respectively. At last, these volumes of abnormal ROIs and the overlapping brain regions in three atlases were adopted as the classification features for the proposed support vector machine (SVM) based classification algorithm, respectively, and the leave-one-out cross-validation (LOOCV) was used to estimate the classification performance.

## Materials and Methods

### Participants

Sixty-nine MCI patients and 63 HC participated in this study, and all participants have not taken any medication that may influence cognition function. All MCI patients were diagnosed by two experienced neurologists, and the detailed inclusion criteria for MCI patients included: (1) memory complaint, confirmed by patient-self or family members; (2) objective memory impairment, adjusted for education and age; (3) normal or near normal activities of daily living; (4) normal or near-normal performance on cognitive function; (5) clinical dementia rating (CDR) score equals 0.5; (6) without dementia according to DSM-IV (Diagnostic and Statistical Manual of Mental Disorders, 4th edition, revised). The 63 HC matched well with MCI patients on gender, age, and education level, and the detailed inclusion criteria for HC included: (1) no nervous system diseases that could cause cognitive function impairment, such as Parkinson’s disease, depression, encephalitis, and brain tumors; (2) no history of psychosis or congenital mental growth retardation; (3) no medication conditions that may interfere with cognitive performance; (4) no visible vascular lesions on sMRI; (5) no history of stroke or dependence on alcohol; (6) no other systemic diseases that cause cognitive impairment, such as syphilis, severe anemia, and HIV. All participants underwent a standardized clinical assessment protocol including mini-mental state exam (MMSE), CDR, and Auditory Verbal Learning Test. Written informed consent forms were obtained from all participants, and this study was approved by the medical research ethics committee of Nanfang Hospital affiliated to Southern Medical University. The detailed demographics and clinical characteristics of all participants were presented in Table 1.

**Table 1:** Participants’ demographic and clinical characteristics.

Characteristics	MCI	HC	*P-*values
Gender (M/F)	69(30/39)	63(27/36)	0.94^#^
Age (years)	66.64 ± 7.70	64.22 ± 7.38	0.07^*^
Education (years)	9.75 ± 4.37	9.35 ± 4.20	0.59^*^
CDR	0.5	0	0^∗^
MMSE	23.03 ± 3.10	27.92 ± 1.58	<0.001^*^
AVLT-immediate recall	8.22 ± 2.54	13.48 ± 3.02	<0.001^*^
AVLT-delay recall	3.68 ± 3.16	10.27 ± 2.57	<0.001^*^
AVLT-recognition	6.49 ± 3.50	11.71 ± 2.32	<0.001^*^

*Values are mean ± SD unless the SD was not calculated. M, male; F, female. CDR, Clinical Dementia Rating scale; MMSE, Mini-Mental State Examination; AVLT, Auditory Verbal Learning Test. ^#^The P-value was obtained by Chi-square test. ^∗^The P-values were obtained by two-sample two-tailed t-test.*

### Data Acquisition

All participants were scanned on a 3.0-Tesla Siemens scanner with 8-channel radio frequency coil at Nangfang hospital. Sagittal structural images for all participants were collected using a magnetization prepared rapid gradient echo (MPRAGE) three-dimensional T1-weighted sequence with the following parameters: repetition time (TR) = 1900 ms, echo time (TE) = 2.2 ms, flip angle (FA) = 9°, inversion time (TI) = 900 ms, matrix = 256 × 256, number of slices = 176, thickness = 1.0 mm, and voxel size = 1 × 1 × 1 mm^3^.

### Image Analysis

All sMRI data were performed with the VBM toolbox (VBM8^1^) implemented in SPM8 according to the following steps: First, the T1-weighted images were checked by two experienced neuroradiologists, and no obvious abnormalities or artifacts were observed in all subjects. Then all images were segmented into gray matter, white matter, and cerebrospinal fluid (CSF) by utilizing the “New-segment” routine in SPM8. Next, all the segmented images were normalized into the Montreal Neurological Institute (MNI) space using the high-dimensional DARTEL normalization algorithm, and the normalized images were modulated with Jacobian matrices to preserve the actual amounts of a tissue class within each voxel. At last, the modulated images were smoothed with an 8-mm full width at half-maximum Gaussian kernel.

### Feature Calculations and Selections Under Three Atlases

**FIGURE 1:**
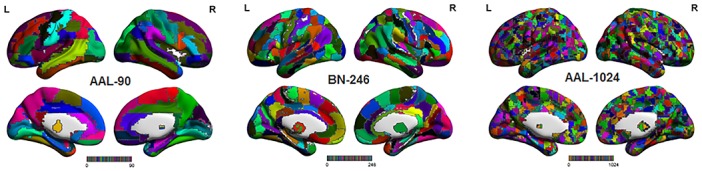
The three atlases including AAL-90 atlas, BN-246 atlas, and AAL-1024 atlas.

The processed sMRI images were utilized to extract the volume features for identifying MCI from HC with three different brain parcellation atlases: AAL-90, BN-246, and AAL-1024 atlas (Figure 1). The AAL-90 atlas, which was generated from 27 high resolution T1-weighted images of a young male (Tzouriomazoyer et al., 2002), partitions the whole cerebral cortex into 90 ROIs (without cerebellum) (Dai et al., 2012; Khazaee et al., 2016). The newly built BN-246 atlas, which was generated based on anatomical connections, divides the whole cerebral cortex into 210 cortical and 36 subcortical subregions (Fan et al., 2016). The AAL-1024 atlas, which is generated by subdividing each region of the low resolution AAL-90 atlas into a set of subregions, partitions the whole cerebral cortex into 1024 ROIs, and every ROI of AAL-1024 atlas has approximately identical size (Wu et al., 2013).

According to the above-mentioned three different parcellation schemes, the volume of each ROI was calculated for all subjects by using the MATLAB program^2^, and the extracted volume features of all subjects in three atlases were served as the candidate features, respectively. Given some features were redundant and irrelevant for classification; it is desirable to select out the discriminative features to improve the classification performance. Several previous studies have demonstrated that properly reducing the number of features can not only improve the performance of the classifier but also speed up the computation (Dosenbach et al., 2010; Dai et al., 2012). Therefore, two-sample two-tailed *t*-test was performed on the candidate features of three atlases, respectively, to determine the significantly different features (*P* < 0.01, uncorrected) as the classification features. Besides, a Fisher score method was also used for feature selection (Khazaee et al., 2016), and the selected features with this method were consistent with two-sample two-tailed *t*-test. The Fisher score criteria for each feature is defined as:

(1) $$FS=\frac{n_{1}{\left(m_{1}-m\right)}^{2}+n_{2}{\left(m_{2}-m\right)}^{2}}{n_{1}\sigma_{1}^{2}+n_{2}\sigma_{1}^{2}}$$

Here *n*_1_ and *n*_2_ represent the number of samples on each group, *m*_1_ and *m*_2_ represent the respective mean value of the feature, m represents the mean value of the total features, and $\sigma_{1}^{2}$ and $\sigma_{1}^{2}$ represent the respective variances. At last, it is worth noting that the feature selection process was only carried out on the training set of each LOOCV fold, which can reduce the overfitting of the classifier.

### SVM-Based Classification Method

The SVM algorithm conceptually implements the idea that the classification features are nonlinearly mapped into a high dimensional feature space where a hyperplane with maximum margin is created to separate the two-group data (Magnin et al., 2009). The SVM algorithm has been widely utilized in neuroimaging studies for its powerful classification performance as well as the simplicity of its theory and implementation (Moradi et al., 2015). In this paper, the LibSVM toolbox^3^ was used to implement the classification.

**FIGURE 2:**
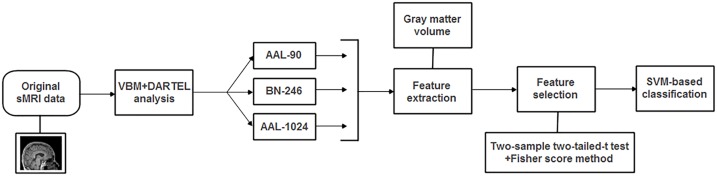
The flowchart of the proposed method for MCI discrimination.

The radial basis function (RBF) defined as (*X,X*_*i*_) → *K*(*X,X*_*i*_) = *e*^*γ*|*X-X*_*i*_|^2^^ was adopted as the kernel function. Besides, in order to improve the classification performance, a grid-search method was utilized to optimize two parameters: γ, the width of the RBF, and C, the penalty parameter of the error term, which adjusts the importance of the separation error in the creation of hyperplane. In this paper, the ranges of these two parameters were γ = 2^-8^,2^-7.5^,...,2^8^ and C = 2^-8^,2^-7.5^,...,2^8^. In detail, the optimal two parameters of γ and C were determined with an internal LOOCV that was only performed on selected features of the training data. The set of parameters obtained the best performance in the internal LOOCV were utilized to train the classification algorithm. In addition, by applying an external LOOCV, the performance of classification method was estimated with the accuracy, sensitivity and specificity, which represent the correctly classified rate of all samples, MCI patients and HC, respectively. It is worth noting that the feature selections and parameter optimization process were only performed on the training set, which could avoid the overfitting of the classifier. In addition, the flowchart of the proposed method for MCI discrimination was shown in Figure 2.

## Results

### Between-Group Differences in Gray Matter Volumes

**FIGURE 3:**
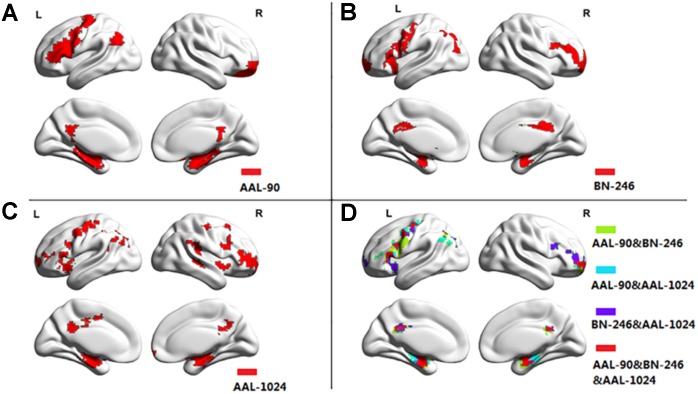
The atrophic brain regions in three atlases, respectively. **(A)** The abnormal brain regions in AAL-90; **(B)** the abnormal brain regions in BN-246; **(C)** the abnormal brain regions in AAL-1024; **(D)** the overlapping abnormal regions among atlases.

**FIGURE 4:**
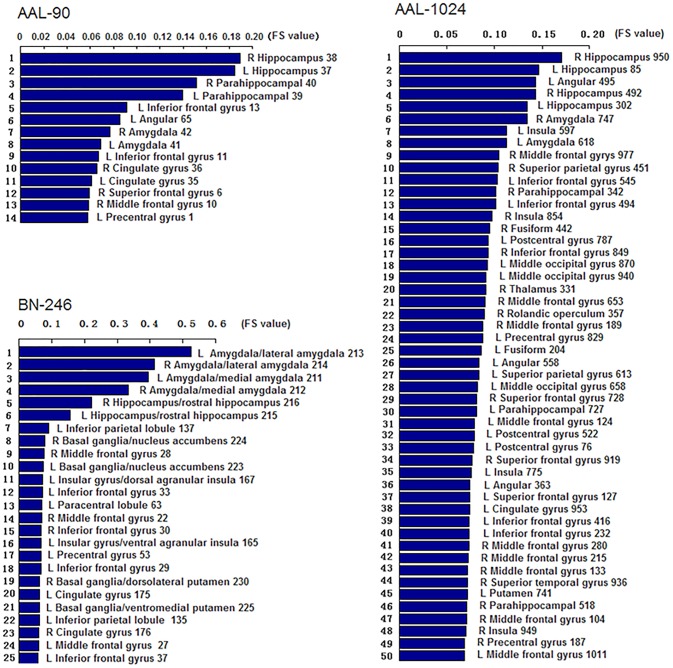
The Fisher score values of the discriminative features in AAL-90, BN-246, and AAL-1024, respectively (The number of the discriminative features in AAL-1024 atlas was 93, and only the prior 50 features were displayed).

Figure 3 showed the ROIs with reliable and discriminative powers during classification process, namely, the features retained more than 125 (132 × 95%, 132 is the total number of cross validation) times in the whole LOOCV process were displayed in the brain mappings. The overlapping abnormal brain regions in three atlases were predominantly located at bilateral hippocampus, bilateral parahippocampal, bilateral amygdala, bilateral cingulate gyrus, left angular gyrus, right superior frontal gyrus, right middle frontal gyrus, left inferior frontal gyrus, and left precentral gyrus. Besides, the Fisher score values of the abnormal ROIs in these atlases were shown in Figure 4.

### Classification Performances Under Three Atlases

**FIGURE 5:**
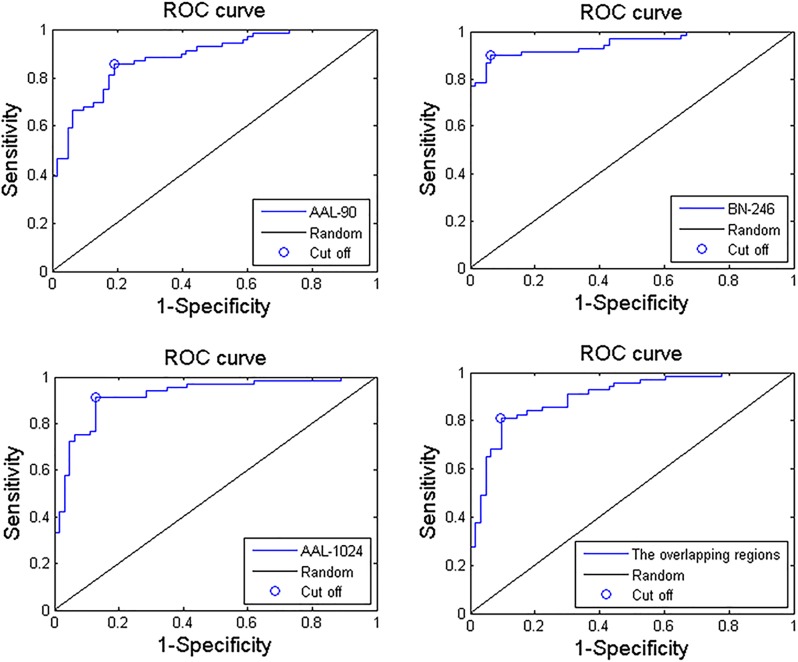
Three ROC curves of the proposed MCI identification method with AAL-90, BN-246, AAL-1024 atlas, and the overlapping abnormal regions, respectively.

When adopting the AAL-90 atlas, a correct classification rate of 83%, a sensitivity of 85%, and a specificity of 81% were obtained. When adopting the BN-246 atlas, a correct classification rate of 92%, a sensitivity of 90%, and a specificity of 94% were obtained. When adopting the AAL-1024 atlas, a correct classification rate of 89%, a sensitivity of 91%, and a specificity of 87% were obtained. When using the volume features of the overlapping abnormal brain regions in three atlases, an accuracy rate was 86%, and sensitivity of 81%, and a specificity of 90% were obtained. The detailed results were summarized in Table 2. Besides, four receiver operating characteristics (ROC) curves were obtained (Figure 5), and the areas under ROC curves (AUCs) with AAL-90, BN-246, AAL-1024 atlas and the overlapping brain regions were 0.89, 0.95 and 0.92, and 0.90, respectively.

**Table 2:** The classification performance of the proposed method on three atlases.

Three atlases	Accuracy	Sensitivity	Specificity
AAL-90	83%	85%	81%
BN-246	92%	90%	94%
AAL-1024	89%	91%	87%
The overlapping regions	86%	81%	90%

## Discussion

This study focused on comparing the classification performance of identifying MCI patients from HC subjects with VBM under three widely used brain atlases, and found that the performance varied in different brain atlases. The best recognition performance was obtained by BN-246 atlas with an accuracy of 92%, indicating a powerful discriminative ability for MCI patients.

**FIGURE 6:**
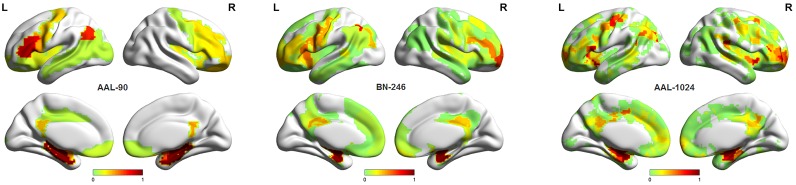
The probability mappings of the selected abnormal features in permutation test with three different atlases.

**FIGURE 7:**
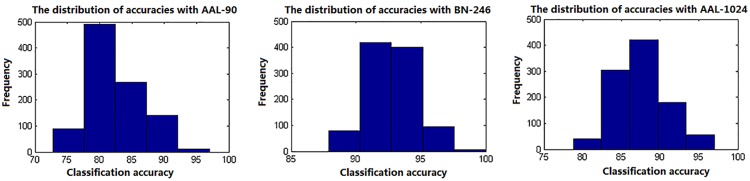
The classification accuracies distributions of the permutation test with three different atlases.

In this paper, a RBF kernel function that could deal with the nonlinear relationship between features and labels was adopted to improve the classification performance (Hsu et al., 2003). The grid search method, which has a high learning accuracy and could be implemented with parallel processing, was utilized to optimize the two parameters of SVM, and it could also improve the classification performance. Besides, considering the feature selections and parameters optimization process were only constrained on the training set of each LOOCV fold, which could reduce the overfitting of the classifier. Thus, the improved accuracies we obtained may be unlikely the inflated accuracies due to overfitting. In addition, the total 90, 246, and 1024 features with parameters optimization were also tested for classification, respectively, and the accuracies without feature selection were all less than 70%, which were significantly lower than that with feature selection. Besides, when using the proposed method to identify MCI patients by extracting the volume features of the overlapping abnormal brain regions in three atlases, the accuracy was 86%. To our best knowledge, it is the first time to classify MCI patients from HC subjects by using the overlapping brain regions in three different atlases. Furthermore, the linear kernel based SVM method and the logistic regression classifier were also applied to the same data to identify MCI patients, and the former classifier achieved accuracies of 80%, 91%, and 87% with AAL-90, BN-246, and AAL-1024 atlas, respectively, and the latter one acquired accuracies of 70, 84, and 76% with AAL-90, BN-246, and AAL-1024 atlas, respectively, suggesting that the RBF kernel based SVM method performed better than these two classifiers and the BN-246 atlas could persistently provide more effective information in identifying MCI patients. Moreover, to validate whether the between-group differences and the performance were stable, the re-sampling based permutation test was performed, which was similar with some previous studies to testify the stability of the between-group differences and classification performance (Magnin et al., 2009; Awate et al., 2016; Awate et al., 2017). In detail, 75% random selected subjects of each group were used to determine the abnormal features and then to train the proposed classification method, then the remaining 25% participants were utilized to estimate the classification accuracy. The above-mentioned processes were repeated 1000 times, and the final accuracies were estimated with the mean of the 1000 re-samplings. The probability of each feature selected in permutation test was defined as the selected times of the feature in the whole process divided by the re-sampling times, and the probability mappings of these selected features were shown in Figure 6. We found that the frequently selected regions in the whole permutation test were consistent with our proposed method. Besides, the accuracies distributions of the permutation test in three atlases were shown in Figure 7, and the final mean accuracies were 82.58, 92.52, and 87.60% with AAL-90, BN-246, and AAL-1024 atlas, respectively, which again demonstrated that our results were stable. At last, the atrophic brain regions in MCI detected by VBM procedure in our study were consistent with many previous VBM studies (Hirata et al., 2005; Hämäläinen et al., 2007; Matsuda, 2013), including hippocampus and parahippocampal, etc. The correspondence indicated the validity of the MRI data and the method of VBM analysis.

In our previous study (Jing et al., 2017), the AAL-90 and AAL-1024 atlas were utilized to make a comparison in identifying major depressive disorder from HC using the functional characteristic, and the AAL-1024 obtained better performance than the AAL-90 atlas. In addition, considering the newly built BN-246 atlas contains both functional and structural connectivity information, thus these three atlases were simultaneously selected in this paper. Through comparing the classification results among them, we found that the BN-246 atlas obtained the best recognition rate than AAL-90 and AAL-1024 atlas. The main reasons for resulting in a considerable disparity in the classification performance may be attributed to the differences between atlases. The AAL-1024 atlas is generated from AAL-90 atlas, and therefore a comparison between BN-246 atlas and AAL-90 atlas is essential for the interpretation of the differences in classification performance.

Brain atlases could be classified into two categories: single-subject topological atlases and population-based probabilistic atlases (Cabezas et al., 2011; Arslan et al., 2017). The AAL-90 atlas is a single-subject atlas generated from a young male (Tzouriomazoyer et al., 2002), whereas the BN-246 is a probabilistic atlas based on 40 MRI data of healthy adults (Fan et al., 2016). This difference might be the major factor resulting in the discrepant classification performance. Namely, no single brain could represent the population due to the neuroanatomical variability across individuals (Devlin and Poldrack, 2007). In addition, the AAL-90 atlas has been found with some other problems such as anatomical variation and methodological limitation. Regarding to the anatomical variation, the AAL-90 atlas displays an atypical rightward asymmetry of planum temporale (PT) that is a triangular structure located on the superior temporal gyrus, and the PT that is involved in mediating sensorimotor control processing has extensive connections to other brain regions (Zheng, 2009). A previous study found microanatomical changes in cortical minicolumn organization of the association cortex in the PT in MCI and AD (Chance et al., 2011), and another previous VBM study also found the early atrophic changes in superior temporal gyrus in AD (Karas et al., 2004). In terms of methodological limitation, the AAL-90 atlas was originally intended to provide a standard reference of anatomical location for functional neuroimaging studies with low spatial resolution (Tzouriomazoyer et al., 2002). However, the partition pattern of AAL-90 does not match the cytoarchitectonic borders well in most cases due that the sulcal and gyral patterns are extremely variable (Amunts et al., 2007). Therefore, the single-subject AAL-90 atlas could not well represent the partition pattern of human brain. Regarding to BN-246 atlas, this atlas partitioned the human brain into distinct subregions based on local structural connectivity architecture, namely, the BN-246 atlas is created by identifying subregions that are maximally homogeneous internally and maximally different from each other in terms of their structural connections (Fan et al., 2016). To some extent, the BN-246 atlas not only confirmed some differentiation from early cytoarchitectonic mappings but also revealed many anatomical subdivisions which were not described previously (Fan et al., 2016). In addition, it is worth noting that although BN-246 atlas showed better classification performance than AAL-90 and AAL-1024 atlas, the BN-246 atlas might not be the best choice, and future neuroimaging studies should pay more attention to the choice of brain parcellation atlases in atlas-based studies.

Another factor that may affect the recognition performance is the number of ROIs in three atlases. Different numbers of ROIs resulted in distinct feature vectors, and the variation in topological patterns of feature vectors corresponded to diverse hyperplanes in feature space, which naturally brought about the discrepancies in the classification performance. In this paper, the performance of AAL-1024 was better than AAL-90 atlas, which may be attributed to the reason that the AAL-1024 atlas could detect more fine abnormalities due to a more subtle parcellation scheme compared to AAL-90 atlas. At last, we found that the Fisher Score value of the volume features with BN-246 was significantly bigger than that of AAL-90 and AAL-1024, which complementarily supported the fact that the BN-246 atlas would obtain the best classification performance in the identification of MCI.

Two issues need to be addressed in this paper. First, some other brain atlases exist in the area of neuroimaging study nowadays, and these atlases could also be utilized to investigate the brain abnormalities affected by atrophy in MCI patients. Second, all the selected atlases in this study did not include cerebellum which may provide some contribution for discriminating MCI patients from HC, and future identification studies could adopt some cerebellum-included atlases to identify MCI patients.

## Ethics Statement

All procedures performed in studies involving human participants were in accordance with the latest Declaration of Helsinki and that all procedures were carried out with the adequate understanding and written consent of the subjects. The study was approved by the ethics committee of Nanfang Hospital.

## Author Contributions

ZhL and JH made substantial contributions to the conception, design, analysis, and interpretation of data and drafted the manuscript. BJ and ZhL made contributions to the revision of the manuscript. BL, ZuL, and ZiL made contributions to the conception, design, and revision of figures. ZhL and HC made contributions to the data acquisition. BJ and HC, the corresponding authors, made contributions to conception and interpretation of data and determined the final version to be submitted for publishing. All authors read and approved the final manuscript.

## Conflict of Interest Statement

The authors declare that the research was conducted in the absence of any commercial or financial relationships that could be construed as a potential conflict of interest.

## Footnotes

1 http://dbm.neuro.uni-jena.de/vbm.html

2 http://www.cs.ucl.ac.uk/staff/G.Ridgway/vbm/get_totals.m

3 http://www.csie.ntu.edu.tw/tildecjlin/libsvm
